# Opportunities and challenges in machine learning‐based newborn screening—A systematic literature review

**DOI:** 10.1002/jmd2.12285

**Published:** 2022-03-23

**Authors:** Elaine Zaunseder, Saskia Haupt, Ulrike Mütze, Sven F. Garbade, Stefan Kölker, Vincent Heuveline

**Affiliations:** ^1^ Engineering Mathematics and Computing Lab (EMCL), Interdisciplinary Center for Scientific Computing (IWR) Heidelberg University Heidelberg Germany; ^2^ Data Mining and Uncertainty Quantification (DMQ) Heidelberg Institute for Theoretical Studies (HITS) Heidelberg Germany; ^3^ Division of Child Neurology and Metabolic Medicine, Center for Child and Adolescent Medicine Heidelberg University Hospital Heidelberg Germany

**Keywords:** data mining, data preprocessing, data science, deep learning, machine learning, modeling, neonatal screening, pattern recognition

## Abstract

The development and continuous optimization of newborn screening (NBS) programs remains an important and challenging task due to the low prevalence of screened diseases and high sensitivity requirements for screening methods. Recently, different machine learning (ML) methods have been applied to support NBS. However, most studies only focus on single diseases or specific ML techniques making it difficult to draw conclusions on which methods are best to implement. Therefore, we performed a systematic literature review of peer‐reviewed publications on ML‐based NBS methods. Overall, 125 related papers, published in the past two decades, were collected for the study, and 17 met the inclusion criteria. We analyzed the opportunities and challenges of ML methods for NBS including data preprocessing, classification models and pattern recognition methods based on their underlying approaches, data requirements, interpretability on a modular level, and performance. In general, ML methods have the potential to reduce the false positive rate and identify so far unknown metabolic patterns within NBS data. Our analysis revealed, that, among the presented, logistic regression analysis and support vector machines seem to be valuable candidates for NBS. However, due to the variety of diseases and methods, a general recommendation for a single method in NBS is not possible. Instead, these methods should be further investigated and compared to other approaches in comprehensive studies as they show promising results in NBS applications.

**SYNOPSIS**

Machine learning can help to further improve newborn screening programs by reducing the false positive rate and hereby increasing specificity and the positive predictive value as well as identifying so far unknown metabolic patterns within the data.

## 1 INTRODUCTION

**FIGURE 1:**
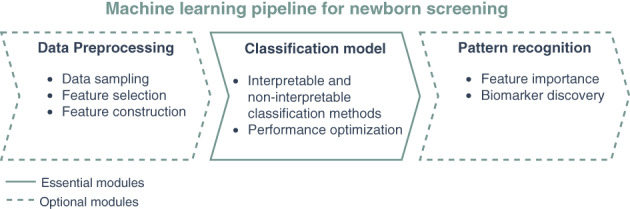
Illustration of machine learning pipeline in NBS. The classification model is the essential part of the ML pipeline in NBS including the interpretable and noninterpretable classification methods and their performance optimization. Data preprocessing is an optional module applied before the classification model. It can include data sampling, feature selection and feature construction methods. Pattern recognition is applied after the classification method evaluating feature importance for biomarker discovery. ML, machine learning; NBS, newborn screening

For more than 50 years, newborn screening (NBS) programs aim at early, ideally presymptomatic, identification of treatable rare diseases with significant health burden to reduce morbidity and mortality. With the introduction of tandem mass spectrometry (MS/MS)^1^, ^2^ and recently genetic methods, NBS panels expanded worldwide^3^, ^4^ and include many inherited metabolic diseases as well as endocrine, hematological, immune and neurological disorders, and cystic fibrosis. NBS programs refer to the screening principles of Wilson and Jungner^5^ which demand a very high sensitivity (ideally 100%) to avoid false negatives and very high specificity (at least 99.5%) to keep the number of false positives low. This is especially challenging in NBS because birth prevalences of the target diseases are very low (1:10 000–<1:1 000 000).^6^ Traditional cut‐off‐based approaches in NBS integrate only a fraction of the available information and focus on the primary variables of the metabolic pathway affected in a particular metabolic disease. Here, laboratory physicians are needed to evaluate these findings and workload directly depends on the number of false positives. Moreover, cut‐off‐based methods cannot deal with complex relationships among metabolites.^7^ To improve the diagnostic specificity of NBS programs an increasing number of second and multiple tier strategies have been developed combining different biochemical^8^, ^9^ as well as biochemical and genetic methods.^10^, ^11^ In contrast to these analytical improvements, mathematical‐based methods are still rarely used to exploit the complete information of NBS test results to improve specificity and positive prediction of NBS. Thanks to advances in data mining and machine learning (ML) as well as the computing landscape in recent years, new opportunities have been created to examine large datasets with high dimensional feature spaces by implementing a ML pipeline for NBS (Figure 1). ML‐based NBS aims at building a classification model, which is part of the essential *classification models* module to predict the outcome of unknown test data and reduce the number of false positive classifications. The high data imbalance caused by the low birth prevalences of the target diseases makes this task very challenging. Thus, often data preprocessing methods such as data sampling, feature construction, and feature selection are applied before classification.^12^ Furthermore, pattern recognition techniques help to detect hidden metabolic interactions within the data.^13^ Hence, the goal of this systematic literature review is to present and evaluate current approaches of ML‐based NBS, to find an overall consensus on its applicability, and to provide future research directions.

## 2 METHODS

This study was conducted and reported according to the preferred reporting items for systematic reviews and meta‐analyses (PRISMA) guidelines (www.prisma-statement.org).

### 2.1 Research questions

The primary outcome of this systematic literature review was to assess the applicability, advantages and limitations of ML‐based NBS. Therefore, we analyzed published studies according to the following questions:

• *Which diseases were investigated in NBS?*

• *Which data preprocessing methods have been applied in NBS?*

• *Which ML classification algorithms have been applied in NBS and how did they perform?*

• *How were pattern recognition techniques implemented in NBS?*

### 2.2 Search strategy

A two‐stage search procedure was conducted to compile relevant papers. In the initial phase, five electronic databases (ScienceDirect, IEEE, ACM, Sage, and PubMed) were searched in May 2021 and October 2021 to collect literature. The search keywords were Newborn Screening AND (Machine Learning OR Deep Learning OR Data Mining). In the second phase, cross‐references from eligible literature of the first phase were searched via Google Scholar and expert advice was added to compile the final literature collection.

### 2.3 Inclusion and exclusion criteria for study selection

All included studies applied an ML classification method in advanced NBS and were published between January 2000 and September 2021. Studies were excluded if they do not concern NBS, do not use data obtained from MS/MS, or do not apply ML algorithms for disease classification.

### 2.4 Study eligibility

Duplicates were removed before assessment. First, titles and abstracts were screened and studies not relating to the research question were excluded. Then, full‐text articles were reviewed for inclusion. In case of exclusion, the reason was reported.

### 2.5 Data extraction and synthesis

Data from all studies, including information on authors, data preprocessing, classification models, performance, and pattern recognition were extracted and summarized in Table 1. For the data analysis we consider key indicators based on their underlying approaches, data requirements, interpretability on a modular level, and performance. The classification performance was evaluated based on the sensitivity, specificity, and positive predictive value (PPV) as summarized in Table 2. These were compared to reference values and other ML methods in comparative studies. For studies lacking sensitivity or specificity values, we calculated these based on the published contingency tables. The studies were insufficient for a meta‐analysis, hence, the findings were synthesized into an overall narrative.

**TABLE 1:** Summary of all reviewed studies on applied data imbalance, feature construction, feature selection and ML classification methods

Author	Disease	Data imbalance	Feature construction	Feature selection	ML classification
Baumgartner et al.^13^	PKU	Random sampling		Information gain	DT, LRA
Baumgartner et al.^14^	MCADD, PKU	Random sampling		Information gain, relief‐based	LDA, DT, KNN, LRA, NN, SVM
Baumgartner et al.^15^	3‐MCCD*, MCADD, PKU	Random sampling		Diagnostic flag	DT, LRA
Baumgartner et al.^16^	3‐MCCD*, PKU, GA1, MMA, PA, MCADD, LCHADD	Random sampling		Discriminatory threshold	KNN, LRA, Naive Bayes, NN, SVM
Ho et al.^12^	MCADD	Informed sampling	Arithmetic ratio	*χ* ^2^	Rule learner
Hsieh et al.^17^	MMA			Pearson coefficient	SVM
Hsieh et al.^18^	MMA	Random sampling		Pearson coefficient	SVM
Van den Bulcke et al.^19^	MCADD	Oversampling	Arithmetic ratio	Variable set optimization	DT, LRA, Ridge‐LRA
Chen et al.^20^	PKU			Fisher score	SVM
Chen et al.^21^	3‐MCCD*, PKU, MET		Arithmetic ratio	Fisher score, Variable set optimization	SVM
Lin et al.^7^	CIT1, CIT2, CPT1D, GA1, IBDD, IVA, MADD, MET, MMA, MSUD, PA, PKU, PTPSD, SCADD*, VLCADD	Random sampling, oversampling, informed sampling		*χ* ^2^, ANOVA, mutual information, L1‐norm, tree‐based	Bagging, Boosting, DT, KNN, LDA, LRA, RF, SVM
Peng et al.^22^	MMA	Oversampling			RF
Wang et al.^23^	SCADD*, MCADD, VLCADD		Arithmetic ratio	Discriminatory threshold	LRA
Zarin Mousavi et al.^24^	CH			*χ* ^2^, information gain, expert consultation	Bagging, Boosting, DT, NN, SVM
Peng et al.^25^	GA1, MMA, OTCD, VLCADD	Second tier			RF
Zhu et al.^26^	PKU		Arithmetic ratio	Pearson coefficient, LVQ	LRA
Lasarev et al.^27^	CAH	Informed sampling	PCA		DT

*Note*: Diseases with * are biochemical variations nowadays known as nondiseases.

Abbreviations: CAH, congenital adrenal hyperplasia; CH, congenital hypothyroidism; CIT1, citrullinemia type I; CIT2, citrullinemia type II; CPT1D, carnitine palmitoyltransferase I deficiency; DT, decision tree; GA1, glutaric aciduria type I; IBDD, isobutyryl‐CoA dehydrogenase deficiency; IVA, isovaleric aciduria; KNN, K‐nearest neighbors; LCHADD, long‐chain hydroxyacyl‐CoA deficiency; LDA, linear discriminant analysis; LRA, logistic regression analysis; LVQ, learned vector quantization; MADD, multiple acyl‐CoA dehydrogenase deficiency; MCADD, medium‐chain acyl‐CoA dehydrogenase deficiency; 3‐MCCD, 3‐methylcrotonyl‐CoA carboxylase deficiency; MET, hypermethioninemia; MMA, methylmalonic aciduria; MSUD, maple syrup urine disease; NN, neural network; OTCD, ornithine transcarbamylase deficiency; PA, propionic aciduria; PCA, principal component analysis; PKU, phenylketonuria; PTPSD, 6‐pyruvoyl‐tetrahydrobiopterin synthetase deficiency; RF, random forest; Ridge‐LRA, logistic ridge regression; SCADD, short‐chain acyl‐CoA dehydrogenase deficiency; SVM, support vector machine; VLCADD, very long‐chain acyl‐CoA dehydrogenase deficiency.

**TABLE 2:** Sensitivity, specificity and positive predictive value (PPV) of considered ML classification methods

Disease	ML classification	Sensitivity (%)	Specificity (%)	PPV (%)	Author
(A) Comparative ML classification studies					
PKU	LRA	100	99.793	17.41	Baumgartner et al.^14^
	LRA	98.0	99.9	–	Baumgartner et al.^13^
	LRA	96.809	99.905	49.46	Baumgartner et al.^15^
MMA	NN	98.0	–	98.0	Baumgartner et al.^16^
MCADD	Ridge‐LRA	100	99.987	33.90	Van den Bulcke et al.^19^
	LRA	96.83	99.992	88.41	Baumgartner et al.^14^
	LRA	95.238	99.992	88.24	Baumgartner et al.^15^
3‐MCCD*	LRA	95.455	99.957	33.33	Baumgartner et al.^15^
CH	Bagging‐SVM	73.33	100	–	Zarin Mousavi et al.^24^
CIT2, MET, MMA, PKU, SCADD*	SVM	91.30	36.36	19.29	Lin et al.^7^
(B) Single ML classification studies					
PKU	SVM	100	99.997 (99.971)	–	Chen et al.^21^
	SVM	100 (100)	99.98 (99.96)	–	Chen et al.^20^
	LRA	97.66	31.61	24.59	Zhu et al.^26^
MMA	SVM	100 (100)	100 (99.79)	–	Hsieh et al.^18^
	RF	100 (100)	89.678 (81.226)	26.40 (16.40)	Peng et al.^25^
	RF	96.117 (96.117)	*65.143 (28.286)*	28.9 (16.5)	Peng et al.^22^
	SVM	95.9 (81.4)	95.6 (76.2)	–	Hsieh et al.^17^
MCADD	LRA	*100 (100)*	*99.988 (99.924)*	18.2 (3.4)	Wang et al.^23^
	RL	100 (100)	99.901 (98.463)	93.75 (49.18)	Ho et al.^12^
GA1	RF	*100 (100)*	*94.503 (50.751)*	22.30 (3.10)	Peng et al.^25^
3‐MCCD*	SVM	100	99.936 (99.711)	–	Chen et al.^21^
MET	SVM	100	99.986 (99.958)	–	Chen et al.^21^
VLCADD	LRA	*100 (100)*	*100 (100*)	100 (100)	Wang et al.^23^
	RF	*100 (100)*	*92.786 (92.639)*	23.40 (23.10)	Peng et al.^25^
OTCD	RF	*100 (100)*	*99.601 (81.983)*	62.10 (3.50)	Peng et al.^25^
SCADD*	LRA	*100 (100)*	*99.997 (99.974)*	73.3 (22.0)	Wang et al.^23^
CAH	DT	*90.909 (100)*	*100 (87.194)*	66.7 (20)	Lasarev et al.^27^

*Note*: (A) Values of best performing ML classification methods with highest sensitivity and specificity in comparative studies. If presented in the study, these are the results from largest or unknown validation datasets. (B) All results of studies applying a single classification method. If sensitivity and specificity were not stated in the study, the results are calculated based on the published contingency table and given in *italics*. Results in brackets show comparison to traditional NBS, where given. Diseases with * are biochemical variations nowadays none as nondiseases. The results from Lin et al.^7^ are presented in a separate row, since they only report average evaluation results for groups diseases. Most studies applied sampling algorithms, changing the sick‐to‐control ratio, and reduced datasets, such as only including false positive patients from traditional screening. Hence, the performance results and reference values of Table 2 have to be evaluated and compared carefully.

Abbreviations: CAH, congenital adrenal hyperplasia; CH, congenital hypothyroidism; CIT2, citrullinemia type II; DT, decision tree; GA1, glutaric aciduria type I; LRA, logistic regression analysis; MCADD, medium‐chain acyl‐CoA dehydrogenase deficiency; 3‐MCCD, 3‐methylcrotonyl‐CoA carboxylase deficiency; MET, hypermethioninemia; MMA, methylmalonic aciduria; NN, neural network; OTCD, ornithine transcarbamylase deficiency; PKU, phenylketonuria; RF, random forest; RL, rule learner; Ridge‐LRA, logistic ridge regression; SCADD, short‐chain acyl‐CoA dehydrogenase deficiency; SVM, support vector machine; VLCADD, very long‐chain acyl‐CoA dehydrogenase deficiency.

## 3 RESULTS

**FIGURE 2:**
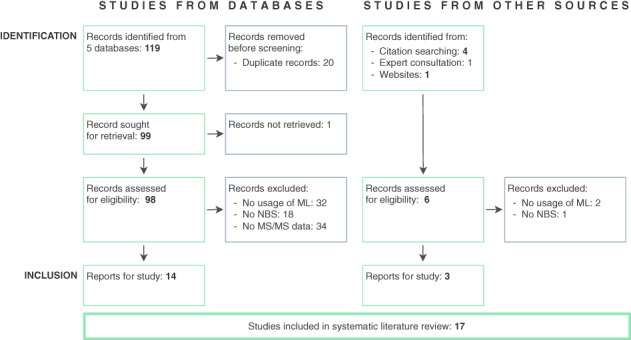
PRISMA flow diagram describing the two‐stage search procedure for studies identified, screened, included, and excluded for this review

Detailed search results of the literature identification process based on the predefined inclusion and exclusion criteria are presented in the PRISMA flow diagram in Figure 2. From the 99 unique publications, we identified 14 as highly relevant. The main reasons for dismissing papers were that they did not apply ML classification methods,^28^ investigated other diseases from NBS programs such as hearing disabilities^29^ or did not use data obtained from MS/MS.^30^ Publications from different screening centers in Europe,^12^, ^13^, ^14^, ^15^, ^16^, ^19^ Asia,^7^, ^17^, ^18^, ^20^, ^21^, ^23^, ^24^, ^26^ and North America^22^, ^25^, ^27^ are reviewed in this work.

### 3.1 Diversity of NBS disease panels

Studies included in the systematic literature review focused on NBS programs for early detection of inherited metabolic diseases and endocrine disorders in newborns, which endanger the physical and mental development of infected children to an extent. Some studies also included biochemical variations nowadays known as nondiseases benign conditions (i.e. short‐chain acyl‐CoA dehydrogenase deficiency, 3‐methylcrotonyl‐CoA carboxylase deficiency). As they were part of the disease panels, they were not excluded from the analysis, but marked as nondiseases in Table 1. The number of diseases included in NBS programs varies over time and depends on the screening center location. In total, 21 diseases were examined in the reviewed studies, whereby only nine were considered in more than one publication (Table 1). From these, phenylketonuria (PKU), methylmalonic aciduria, and medium‐chain acyl‐CoA dehydrogenase deficiency were the most frequently examined (Table 1).

### 3.2 Applied data preprocessing methods

Data preprocessing is usually the first step in the ML pipeline (Figure 1) and deals with preparing and transforming the data into a suitable form for classification algorithms. It includes data imbalance, feature construction, and feature selection methods in NBS (Table 1). All of the evaluated studies applied at least one preprocessing method.

#### 3.2.1 Data imbalance

**FIGURE 3:**
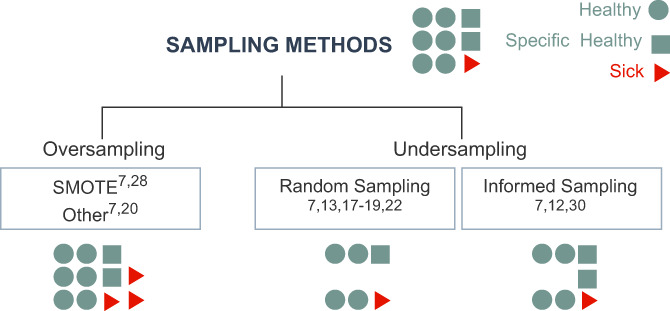
Applied sampling methods. Imbalanced datasets consist of healthy patients (•), special subsets of healthy patients (■), and sick patients (▶). Oversampling adds synthetically created sick patients to the dataset. Undersampling methods reduce the number of data points: random sampling randomly excludes healthy patients, informed sampling excludes only specific subsets of healthy patients. In each box the studies applying the respective method are given

Common methods to overcome data imbalance are sampling methods, which either increase (oversampling) or decrease (undersampling) the data^31^ (Figure 3). In NBS, *informed sampling* is applied to include special subsets of healthy patients. The inclusion is mainly based on clinical criteria such as healthy patients with elevated primary markers,^12^ particularly removing samples close to the decision boundary,^7^ one‐sided selection,^7^ or healthy patients with varying birth weight and gestational age.^27^ Other inclusion criteria are based on Tomek links and edited nearest neighbors.^7^ *Random sampling* is applied to change the data imbalance to ratios, for instance, between 1:4^18^ and 1:25^14^ by randomly excluding data points. In contrast, *oversampling* methods are applied rarely and create synthetic data samples from the minority class by applying randomness or cluster‐based methods such as synthetic minority oversampling technique (SMOTE)^7^ and Borderline‐SMOTE.^7^ Furthermore, spiked blood samples which are designed to resemble sick blood samples are added to enrich the datasets^19^ and mixed models such as SMOTE + ENN^7^ were applied. For studies that applied ML in second tier analysis, the data was less imbalanced since it only contained false positive screening results from the first tier.^25^

#### 3.2.2 Feature construction

Most feature construction methods combine existing numerical features to build new complex features. In NBS, mostly arithmetic operators on original features are used to construct features (Table 1). A new feature $x^{\prime }$ can be built from two features $x_{i},x_{j}$ by calculating their ratio^19^, ^21^, ^23^, ^26^:

$$x^{\prime }=\left[x_{i}/x_{j}\right];\;i=0,1,\ldots ,n-1;\;j=i+1,i+2,\ldots ,n,$$

or by combining several original features.^12^ Here, $x_{i},i=1,\ldots ,n$ can be all original features^12^ or a subset of disease‐specific primary markers.^19^ Other approaches applied principal component analysis,^27^ which computes eigenvectors of the data's covariance matrix, the principal components, which are then used as features or applied self‐developed algorithms^21^ to identify relevant features for feature construction.

#### 3.2.3 Feature selection

**FIGURE 4:**
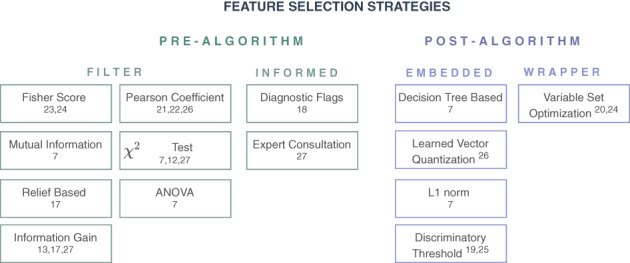
Applied feature selection strategies. Prealgorithm strategies (left) work independent of the ML classification method and are filter methods, using statistical properties, or informed methods, using clinical knowledge. Postalgorithm methods (right) are directly embedded within the classification method or wrapped around it via an iterative loop. In each box the studies applying the respective method are given. ML, machine learning

Feature selection methods aim at identifying the most relevant features and reducing the dimensionality of the feature space. Either a fixed number^18^, ^21^ or adaptive approaches^12^ are applied to decide how many features should be selected. They can be distinguished by their application procedure, before (pre) or after (post) a classification algorithm and are grouped in *filter*, *wrapper*, and *embedded* methods^32^ (Figure 4). For NBS, a fourth category, *informed* methods, was added. Sometimes, several of these methods were applied sequentially^16^, ^26^ (Table 1).

In NBS, filter methods are frequently applied. They select features based on statistical measures and properties such as analysis of variance (ANOVA),^7^ *χ* ^2^ tests,^7^, ^12^, ^24^ mutual information,^7^ Pearson‐like formula,^17^, ^18^, ^26^ Fisher score,^20^, ^21^ information gain,^13^, ^14^, ^24^ and relief‐based methods.^14^ Informed approaches apply prior knowledge obtained from experts or literature to select relevant features. In NBS, these are established diagnostic flags, which are developed by biochemical and medical experts^15^ or important features based on results of consultation with a pediatric endocrinologist.^24^

Embedded methods exploit the architecture of the classification method to understand the impact different features have on its performance. In NBS, decision tree splitting rules,^7^ the discriminatory threshold from logistic regression analysis (LRA),^16^, ^23^ learned vector quantization,^26^ and underlying cost functions, such as L1 norm^7^ were analyzed for feature selection. Wrapper methods choose different subsets of all features^19^ or subsets preselected by another method^21^ and iterate through the algorithm to detect feature combinations which optimize the performance of the classification method.

### 3.3 Application and performance of ML classification algorithms

**FIGURE 5:**
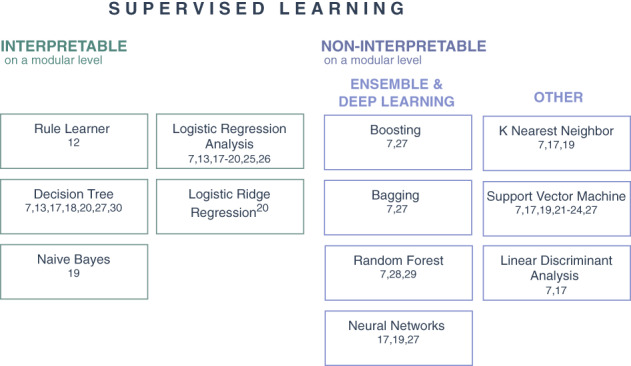
ML classification methods applied in NBS. The methods are distinguished according to their interpretability and functionality. Interpretable methods on a modular level (left) and noninterpretable methods on a modular level (right) which can be split into ensemble and deep learners or other methods. In each box the studies applying the respective method are given. ML, machine learning; NBS, newborn screening

NBS data contain individuals with confirmed diagnosis, hence, only supervised classification methods are applied (Table 1). We grouped these methods according to their interpretability and functionality (Figure 5). There are various definitions of interpretability where we here apply *interpretability on a modular level*, referring to methods that can inherently explain how parts of the model affect predictions.^33^

#### 3.3.1 Interpretable methods

LRA^7^, ^13^, ^14^, ^15^, ^16^, ^19^, ^23^, ^26^ is based on modeling the distribution of discrete dependent features. For instance, the LRA model from Baumgartner et al.^13^ for PKU was stated as

$$P\left(\mathrm{PKU}\right)={\left(1+e^{-0.056\times \mathrm{Phe}+8.9269}\right)}^{-1},$$

where Phe is the amount of the measured phenylalanine concentration and yields the probability of a patient suffering from PKU. Ridge logistic regression analysis (Ridge‐LRA)^19^ extends this method by adding a penalty term to the logistic regression function and is applied when independent features are highly correlated. For both methods, the weights of the resulting regression coefficients are the interpretable part of the model.^33^ Decision trees^7^, ^13^, ^14^, ^15^, ^19^, ^24^, ^27^ are used to subsequently divide the dataset into subsets by applying an impurity index to minimize the impurity of the subsets. They can be interpreted using the splitting decisions and leaf node predictions. Rule learners^12^ classify patients by finding interpretable decision rules which can be applied for classifying unseen datasets. Naive Bayes^16^ is a probabilistic method based on applying Bayes' theorem with strong independence assumptions. It can be interpreted on a modular level by interpreting the conditional probability through estimating how much each feature contributes to a specific classification.

#### 3.3.2 Noninterpretable methods

Ensemble methods combine several weak learners to obtain one strong classification algorithm and are noninterpretable even if the underlying weak learner is interpretable on a modular level. Random forest^7^, ^22^, ^25^ combines several randomly initialized decision trees to obtain one powerful classifier. Boosting algorithms such as adaptive boosting (ADA),^7^, ^24^ extremely randomized trees, and gradient boosting are ensemble meta‐algorithms for primarily reducing bias and variance, where each weak learner tries to correct the model predictions of its predecessors. Furthermore, bagging methods such as Bagging‐SVM^7^, ^24^ were applied as meta‐estimators to train several base‐classifiers on randomly sampled subsets and aggregate the predictions. Neural networks^14^, ^16^, ^24^ try to mimic the signaling processes in the human brain by leading information through multilayer perceptrons. Other methods such as K‐nearest neighbor^7^, ^14^, ^16^ consider the distance between data points and identify clusters of healthy and sick patients within the data. Support vector machines (SVMs)^7^, ^14^, ^16^, ^17^, ^18^, ^20^, ^21^, ^24^ attempt to find the largest separating band between sick and healthy patients by transforming the features into a higher dimensional space with linear kernels,^7^, ^16^ radial basis function,^16^, ^18^, ^20^, ^21^ or polynomial (degree 2 or 3)^14^, ^16^ kernels. Linear discriminant analysis^7^, ^14^ is a linear classifier that aims to find a discriminant line (plane or hyperplane) by fitting weights of features that are optimal for maximizing the between‐class variance.

#### 3.3.3 Performance results

For parameter optimization, grid search is commonly applied^17^, ^19^ and iterates through a set of parameter combinations returning the combination with the best performance. To test the robustness of the methods and estimate the performance on different subsets, cross‐validation^17^, ^21^ or stratified cross‐validation^19^ and evaluation of receiver operating characteristic curves^25^ is applied. The classification performance is evaluated using classification sensitivity, specificity, and PPV, which are calculated using the amount of true positive (TP), false positive (FP), true negative (TN), and false negative (FN) predicted patients,

$$\text{sensitivity}=\frac{\mathrm{TP}}{\mathrm{TP}+\mathrm{FN}},\;\text{specificity}=\frac{\mathrm{TN}}{\mathrm{TN}+\mathrm{FP}},\;\;\mathrm{PPV}=\frac{\mathrm{TP}}{\mathrm{TP}+\mathrm{FP}}.$$

Here, 100% sensitivity reflects finding all sick newborns and an increasing specificity implies fewer false positive patients. PPV expresses the probability that positively predicted patients are truly suffering from a disease. Table 2 gives an overview of all published results from (A) comparative studies, displaying only the results of the best from several applied classification methods and (B) single studies, applying only one classification method.

Hence, from all 12 considered ML classification methods only LRA, Ridge‐LRA, SVM, Bagging‐SVM, rule learner, neural network, random forest, and decision tree are included in the performance evaluation. From these, LRA and SVM were applied most frequently. In the comparative studies, LRA, Ridge‐LRA, SVM, Bagging‐SVM, and neural networks were the best performing algorithms for different diseases. Moreover, 10 studies reported reference values from traditional NBS on the same datasets. In all of these studies, the ML classification improved the PPV compared to the reference values. Furthermore, applying SVM, random forest, LRA, or rule learners maintained or improved the sensitivity, compared to the reference value in every study. While maintaining the high sensitivity, mostly 100%, all of these methods improved the specificity. Overall, for 9 of the 21 evaluated diseases, ML classification methods achieved 100% sensitivity. Lin et al.^7^ presented the most comprehensive study, where SVM, LRA, and linear discriminant analysis showed the best average evaluation results on groups of 5 and 16 diseases. However, when they evaluated single diseases, they showed that also ADA is appropriate for NBS if the dataset includes sufficient patients suffering from a specific disease.

### 3.4 Implementation of pattern recognition techniques in NBS

Pattern recognition techniques are strongly related to feature selection since they aim to recognize patterns within the features' importances. Therefore, the results of the feature selection methods are often compared to established primary markers^7^ and analyzed by clinical and biochemical experts.^15^ Furthermore, for interpretable methods, built‐in decision functions^12^, ^14^, ^15^, ^24^ and discriminatory thresholds^14^, ^16^ can be used to identify the biomarkers on which the classifier based its classification. For noninterpretable ML methods, model agnostic approaches such as mean decrease accuracy are applied to identify the individual contribution of specific metabolites.^22^, ^25^

## 4 DISCUSSION

Further development and optimization of NBS for inherited metabolic diseases remains an important and challenging task. Based on a systematic review, we identified opportunities and challenges of the whole ML pipeline for NBS, including the application of data preprocessing, classification models and pattern recognition methods.

### 4.1 Opportunities

The evaluated studies showed that ML methods can be applied for classifying NBS data and presented high classification sensitivity and specificity on several diseases. They were able to decrease the number of false positives compared to reference values from traditional screening,^14^ to find metabolic markers without prior assumptions which correspond to the established biochemical knowledge,^13^, ^15^ and to identify so far unknown metabolic patterns within the data.^14^ From all included classification methods, LRA, Ridge‐LRA, SVM, Bagging‐SVM, and neural network achieved best results in comparative studies (Table 2A) indicating that these methods outperform others for NBS classification. Results from single method studies (Table 2B) are more difficult to evaluate as a comparison on the specific dataset is missing. We analyzed the performance of the classification methods based on their frequency of application, ability to achieve 100% sensitivity, a comparison with other classification methods, and reference values. From the reported results, LRA and SVM seem to be valuable candidate methods for NBS classification. Both algorithms are frequently applied in NBS, achieve 100% sensitivity for various diseases in several studies, are the best algorithms in most comparative studies, and can increase the sensitivity, specificity, and PPV compared to reference values from traditional screening (Table 2). Also, advanced versions of these methods such as Bagging‐SVM and Ridge‐LRA achieved the best results in two comparative studies.^19^, ^24^ Furthermore, we analyzed their interpretability on a modular level, referring to whether they can inherently explain how parts of the model affect the prediction.^33^ LRA is interpretable, as the model and weights can be intuitively interpreted. The separating hyperplane of SVM is difficult to interpret on a modular level, particularly, when the original variables are embedded into a higher dimensional space with a kernel trick.^14^, ^16^, ^18^, ^20^, ^21^

However, the classification methods should not be evaluated on their own, as they are usually part of a whole ML pipeline (Figure 1) including data preprocessing methods, which can further influence the classification performance. Sampling methods can be applied to decrease false positive classifications utilizing expert knowledge on primary markers. Feature construction methods enable to build complex features, which can account for nonlinear correlations spread over several metabolites and discover hidden interactions.^12^, ^19^, ^23^ This can increase the accuracy of LRA and other classifiers such as rule learner methods, which do not perform well for problems requiring diagonal partitioning.^12^, ^34^

Feature selection techniques are employed to eliminate irrelevant and redundant information for the classification method to reduce dimensionality and overfitting and allow classification algorithms to operate faster and more efficiently.^14^ They can reduce the number of positive NBS results and improve sensitivity and specificity in NBS classification.^13^, ^20^, ^26^ Furthermore, pattern recognition showed great potential by confirming primary diagnostic markers and identifying markers without any other a priori assumptions or conditions.^15^ Model agnostic pattern recognition can be applied for noninterpretable methods by discovering nonexplainable incidents such as a higher percentage of false positive newborns with Hispanic ethnicity.^22^, ^35^ This can be especially beneficial for varying prevalence between racial/ethnic groups and populations.^25^, ^26^, ^35^ Furthermore, these methods can help to identify other risk factors such as *gender*, *family disease history*, and *chronic diseases* to identify infants with potential disease risk.^24^

### 4.2 Limitations and future work

The heterogeneity of the 17 studies, including data from 10 screening centers, investigating 21 diseases, applying 12 classification methods and 14 feature selection strategies (Table 1) makes an evaluation of the results challenging, and requires a careful interpretation.

#### 4.2.1 Preprocessing methods

Sampling methods are a promising approach to handle the data imbalance in NBS. However, oversampling methods could pose a problem since it cannot be verified whether the synthetically created samples correspond to a positive confirmation diagnosis. Moreover, sampling methods artificially change the sick‐to‐control ratio of a patient dataset, which could change the model's accuracy on a real population.^13^, ^19^ Hence, sampling methods should be chosen carefully and evaluated on real populations to verify performance measures in real settings.

Feature selection is applied to support the classification method by identifying relevant features. When deciding which method to choose, several criteria have to be taken into consideration. Prealgorithm methods are independent of the classification method and its respective computational costs. However, they do not take into account the biases of the classifiers which can be problematic when classification methods are highly sensitive to the feature selection procedure.^36^ In contrast, postalgorithm methods depend on the specific biases and heuristics of the classification method. This can make them computationally more expensive, as wrapper methods for instance iterate through subsets of all features. Wrapper methods such as mean decrease in accuracy can also be used to rank the relative importance of individual features in a random forest model for pattern recognition.^22^, ^25^ Furthermore, the applicability for NBS has to be evaluated based on its specific data requirements. NBS has numerical input and categorical output data. However, *χ* ^2^ and mutual information expect a categorical input and Pearson's correlation coefficient expects numerical output values whereas ANOVA expects numerical input and categorical output values, which would be most appropriate for NBS. Informed methods allow to include expert knowledge into the feature selection process which can be beneficial for well‐studied diseases but lowers the chances of discovering new metabolic patterns.

#### 4.2.2 ML classification methods

Most studies applied sampling algorithms, changing the sick‐to‐control ratio, and reduced datasets, such as only including false positive patients from traditional screening. Hence, the performance results and reference values in Table 2 have to be evaluated and compared carefully. Classification methods require a certain minimum amount of data to learn the underlying classification processes depending on the task and data. NBS suffers from data limitations due to the rare true positives which lead to diseases being excluded for ML‐based NBS.^14^ However, first experimental studies showed methods trained with more than 20 positive patients achieve stable results.^7^

Furthermore, in many medical ML applications, noninterpretable methods are state‐of‐the‐art.^37^ In NBS, methods such as ensemble learners and neural networks are applied rarely but could surpass interpretable methods in comparative studies.^16^, ^24^ Reasons for this could be that they are not well‐suited for NBS, or their lack of interpretability makes them less applicable. These points could be addressed in a comparative study including different diseases, classification methods, and datasets, ideally, a benchmark dataset. The results of the study should be analyzed with respect to large variations in parameter settings to estimate the stability of the performance.^19^ The lack of interpretability could be addressed by integrating explainable artificial intelligence methods such as SHAP^38^ and LIME^39^ to explain which metabolites contributed to the algorithm's classification results. Furthermore, the developed ML methods could be applied for future NBS conditions aiming at reducing false negative classification results. Here, feature importance and explainable artificial intelligence methods could be implemented for pattern recognition and could play a key role in identifying so far unknown metabolic patterns within the data. Nevertheless, the proposed biomarkers require further validation and evaluation regarding their biochemical role and underlying biological processes in health and disease.^13^, ^16^ Although ML methods showed great potential in classifying NBS conditions based on screening data, their reliability has to be proven by thorough validation studies to adhere to regulatory and quality requirements before they can be integrated into NBS programs. Here, explainable AI methods, can contribute to enhance reliability of ML methods for clinical integration.

### 4.3 Conclusion

Through technical advances, ML‐based NBS enables new opportunities in reducing false positive rates and identifying so far unknown metabolic patterns by relying on complex feature combinations instead of predefined cut‐off values. These mathematical strategies should be regarded as complementary to the combined use of biochemical and genetic tests aiming at improving the diagnostic specificity of NBS programs through second and multiple tier analysis. However, due to the variety of diseases and methods, a general recommendation for a single ML method in NBS is currently not possible. Instead, a thorough analysis of different methods is necessary for all applications. Among the presented, LRA and SVM seem to be valuable candidates for NBS classification since they are often applied, achieve high performance in general and in comparative studies, and handle multidimensional data. Comparing both methods, LRA is interpretable on a modular level, whereas SVM is not and therefore, LRA might be more applicable for NBS. Yet, with the rise of ensemble and deep learning methods, also noninterpretable extensions of these methods such as Ridge‐LRA and Bagging‐SVM showed promising results.^19^, ^24^ In combination with explainable artificial intelligence methods, these noninterpretable methods could be applied more frequently, which will be investigated in comprehensive future studies.

## CONFLICTS OF INTEREST

The authors declare no conflicts of interest.

## AUTHOR CONTRIBUTIONS

All authors have participated in planning, conducting, reporting, and revision of the manuscript before submission.

## ETHICS STATEMENT

This study did not require ethical approval or informed consent by patients.

## Supporting information


**Supplementary Material 1.** PRISMA checklist for “*Opportunities and Challenges in Machine Learning‐based Newborn Screening ‐ A systematic literature review”*

## Data Availability

This study has no associated data.
